# What pre-deployment and early post-deployment factors predict health function after combat deployment?: a prospective longitudinal study of Operation Enduring Freedom (OEF)/Operation Iraqi Freedom (OIF) soldiers

**DOI:** 10.1186/1477-7525-11-73

**Published:** 2013-04-30

**Authors:** Lisa M McAndrew, Elizabeth D’Andrea, Shou-En Lu, Bhavna Abbi, Grace W Yan, Charles Engel, Karen S Quigley

**Affiliations:** 1 Department of Veterans Affairs, NJ War Related Illness & Injury Study Center, 385 Tremont Ave. Mailstop 129, East Orange, NJ 07018, USA; 2 School of Public Health, University of Medicine & Dentistry of New Jersey, Piscataway, NJ, USA; 3 Philadelphia VA Medical Center, Philadelphia, PA, USA; 4 Department of Psychiatry, F. Edward Hebert School of Medicine, Uniformed Services University, Bethesda, MD 20814, USA; 5 Department of Veterans Affairs, Bedford Memorial Hospital, Bedford, MA, USA; 6 Interdisciplinary Affective Science Laboratory, Northeastern University, Boston, MA, Canada

**Keywords:** Health function, Quality of life, Veterans, Military, Prospective, SF-36, Iraq, Afghanistan, Combat

## Abstract

**Background:**

Physical and mental function are strong indicators of disability and mortality. OEF/OIF Veterans returning from deployment have been found to have poorer function than soldiers who have not deployed; however the reasons for this are unknown.

**Methods:**

A prospective cohort of 790 soldiers was assessed both pre- and immediately after deployment to determine predictors of physical and mental function after war.

**Results:**

On average, OEF/OIF Veterans showed significant declines in both physical (t=6.65, p<.0001) and mental function (t=7.11, p<.0001). After controlling for pre-deployment function, poorer physical function after deployment was associated with older age, more physical symptoms, blunted systolic blood pressure reactivity and being injured. After controlling for pre-deployment function, poorer mental function after deployment was associated with younger age, lower social desirability, lower social support, greater physical symptoms and greater PTSD symptoms.

**Conclusions:**

Combat deployment was associated with an immediate decline in both mental and physical function. The relationship of combat deployment to function is complex and influenced by demographic, psychosocial, physiological and experiential factors. Social support and physical symptoms emerged as potentially modifiable factors.

## 

Health function refers to one’s ability to conduct normal daily activities and fulfill usual roles [1]. Health function is a strong predictor of disability and mortality, even after controlling for objective health outcomes, such as illness status [2-4]. It is also a useful indicator of successful reintegration into civilian life in Operation Enduring Freedom (OEF) and Operation Iraqi Freedom (OIF) veterans [5]. Historically, veterans seeking treatment at Veterans Affairs facilities have poorer functioning than the general population, [6-10] however the reasons for this are unclear. In a non-treatment-seeking sample, Kline et al. found that those deploying to OEF/OIF after a previous deployment, were twice as likely to have poor physical function (i.e., below national norms) as those with no previous deployment, suggesting that deployment adversely impacts function [11]. Other work suggests that OEF/OIF veterans with PTSD and chronic pain have lower physical [12,13] and mental [14,15] function compared to healthy controls.

This study was designed to determine factors related to health function after deployment and fill two important gaps. First, no study has used a prospective design to examine pre-deployment factors related to physical or mental function after deployment. Second, few studies have examined demographic, psychosocial, deployment experiences, and physiological factors contributing to function after deployment. To address these limitations, we report on two waves (pre-deployment, immediate post-deployment) of a prospective longitudinal cohort study of military personnel. Our pre-deployment data permit us to both assess and control for possible baseline individual differences in function. Based on existing cross-sectional literature, we hypothesized that demographic factors such as age, psychosocial factors such as coping skills, physiological factors such as blood pressure reactivity to stressors, and experiential factors such as deployment experiences would be related to physical and mental function immediately after a combat deployment. We also hypothesized that individuals who reported experiencing more post-traumatic stress and physical symptoms also would report lower overall health function, although this was not expected to fully explain poorer function.

With almost 2 million OEF/OIF Veterans having returned from combat, understanding factors contributing to health function after war is critical. This study is a necessary first step toward identifying individuals who may be at increased risk for poor function after deployment, and provides knowledge that will be important for practitioners attempting to reduce poor functional health and disability for veterans.

## Methods

We used a prospective longitudinal observational cohort design to assess soldiers who were preparing for deployment to Iraq or Afghanistan at Fort Dix, NJ or Camp Shelby, MS. Data were obtained at 4 time points: pre-deployment (Phase 1, 2005–2008), immediately post-deployment (Phase 2, 2007–2009), 3 months after return from deployment (Phase 3, 2007–2010), and 1 year after return (Phase 4, 2008–2011). In this report we focus on the factors contributing to physical and mental function immediately after deployment (i.e., we report here only data from Phases 1 and 2). The protocol was approved by Institutional Review Boards of all participating facilities.

### Participants

We recruited 790 Army National Guard and Army Reserve enlisted Soldiers (ages 18–60 years) with testing either during or just after their on-base pre-deployment medical processing. The Phase 1 exclusion criteria were current self-reported depression, taking medications with cardiovascular and/or autonomic effects, history of schizophrenia or bipolar disorder, or current cancer, high blood pressure, or pregnancy. Deployments typically lasted 12–13 months. Four hundred twenty-two soldiers were able to be tracked and completed Phase 2 (53%). Of those who did not complete Phase 2, 23 did not wish to continue and the remaining could not be found.

### Procedures

At Phase 1, soldiers completed the informed consent process, then completed several computerized surveys (20–30 minutes), followed by a stress reactivity protocol (20 minutes) and then the remaining surveys (20–30 minutes). We conducted Phase 2 surveys at the Army installation where possible (45 minutes). We attempted to contact soldiers who did not return to either installation using contact information provided at Phase 1. Those successfully reached through their contact information at home completed the measures via mailed surveys and phone follow-up. Measures that were completed at the military installation were completed using a custom-designed computer program (Labview, National Instruments, Austin, TX), which detected if responses were not within a valid range and prompted for corrections. A research assistant was available to answer questions. At any time, participants with responses to individual survey items that suggested the possibility of severe depression or anxiety were provided referral resources as needed.

### Outcome measure

Our primary outcome measures for these analyses were the Veteran’s Rand-36 (VR-36 [3]) which was derived from the Medical Outcomes Survey Short-Form-36 (SF-36 [16]) a commonly used measure of mental and physical functional status. The VR-36 provides two composite scores, physical function and mental function with higher scores indicating better function. Composite scores are normed to a mean of 50 and a SD of 10.

### Survey measures

At Phase 1 we collected self-report demographic information on age, gender, years of education, number of prior deployments, racial and ethnic identity, body mass index (BMI) and military component (Army National Guard vs. Army Reserve). Racial and ethnic identity was dichotomized as White Non-Hispanic or minority (e.g., African American, Hispanic, etc.). Psychosocial variables measured at Phase 1 were negative emotionality [17], absorption [18] (a measure of the tendency to be fully absorbed in one’s own thoughts), pre-deployment stressful life events [19], social desirability (a measure of the tendency to portray oneself in a favorable light on self-report measures) [20], social support [21], physical symptom severity (a measure of both the number of symptoms and extent to which those symptoms are bothersome) [22] and coping style (approach coping involves active engagement with problems, whereas avoidance coping is avoidance of engagement) [23]. Each of these demographic and psychosocial variables was chosen based on the prior literature as likely to be related to health function. Deployment variables were measured immediately after return from deployment and included: deployment experiences (from the Deployment Risk and Resilience [DRRI] Aftermath of Battle subscale) [19], combat experiences (from the DRRI Combat Experiences subscale) [19], unit cohesion [24], PTSD symptoms [25,26] and physical symptoms [22]. Injury in theater was determined from a single item “Did you have any injuries (including minor injuries or injuries for which you did not seek treatment) during your deployment from any of the following: vehicular accident (including airplane), fall or fight involving a blow to the head, fragment, bullet, blast, other”. Again, we chose these variables as potential predictors of physical and mental function based on prior literature.

At Phase 2 we also asked about smoking status at Phase 1, and years of education. We also again collected data on negative emotionality, social desirability, social support, coping style and physical symptoms.

### Physiological measures

Prior research has suggested that both exaggerated [27] and blunted blood pressure reactivity [28] to laboratory stressors can be related to later problematic health outcomes. Our goal was to determine the impact of blood pressure reactivity to a series of stressors at pre-deployment on physical and mental function post-deployment. Participants completed a set of stress reactivity tasks before deployment. These tasks included a speech planning task and a speech task where participants first planned (4 minutes) what they would say when later required to speak to a friend whom they imagined had stolen money from them. The participant then spent 4 minutes speaking into a microphone in front of a computer monitor as if speaking to the friend. Another task was a 4 minute serial subtraction mental arithmetic task where participants were asked to count backwards by a given subtrahend (e.g., sevens) and informed when they were incorrect, with numbers changed each minute during the task to maintain engagement. The final task was a hand cold pressor task where participants were asked to place their hand in icy cold water for up to 2 minutes. Blood pressure was recorded once per minute for five minutes during a resting baseline (pre-stressor levels) and at one minute intervals during these common laboratory stressors using standard psychophysiological methods [29]. Baseline systolic and diastolic baseline blood pressure values were operationalized as the mean of the five baseline blood pressure readings. The blood pressure response to tasks with strong social evaluation/performance anxiety components (speech planning, speech and math) correlated highly with one another, and served as our measure of blood pressure reactivity. Blood pressure reactivity was operationalized as the mean systolic and diastolic blood pressure during the 12 minutes of the speech planning, speech and math tasks minus the mean baseline blood pressure.

### Data analysis

Descriptive statistics for all measures are reported in Table 1. We also examined the bivariate relationships between all continuous variables using Pearson correlation coefficients. Variables that correlated at p ≤ 0.10 with physical or mental function were used in the two multivariate models predicting physical and mental function, respectively.

**Table 1:** **Measures**, **descriptive statistics and correlations**

**Construct**	**Number of items**	**Cronbach’s alpha**	**Scale range**	**Phase, Mean ± SD**	**Correlation**	**Correlation**	**Correlation**	**Correlation**
					**with VR**-**36**	**with VR**-**36**	**with VR**-**36**	**with VR**-**36**
					**PCS at P1**	**PCS at P2**	**MCS at P1**	**MCS at P2**
VR-36 PCS	36	#	0-100	P1=55.49 ± 5.22	--	.32**	-.15**	-.01
				P2=53.28 ± 7.65	.32**	--	.03	-.25**
VR-36 MCS	36	#	0-100	P1=48.00±9.10	.15*	.03	--	.35**
				P2=44.05±10.76	-.01	-.25**	.35**	--
Negative Emotionality	30	P1=.86	0-30	P1= 9.57 ± 5.99	-.09**	-.06	-.61**	-.41**
Absorption	34	P1=.89	0-34	P1=15.23 ± 7.58	-.03	.00	-.21**	-.15**
Pre-deployment Life Events	17	#	0-17	P1= 5.98 ± 3.59	-.05	-.08*	-.15**	-.09
Social Desirability	20	P1=.76	0-20	P1=12.04 ± 3.84	-.02	-.08	.35**	.29**
Social Support	18	P1=.96	0-100	P1=73.78 ± 18.86	.00	-.02	.27**	.25**
Approach Coping	18	P1=.78	0-18	P1=11.20 ± 2.79	.01	-.10**	.11**	.10**
Avoidance Coping	18	P1=.82	0-18	P1=7.63 ± 3.18	-.02	-.08*	-.47**	-.24**
Deployment Experiences	15	#	0-15	P2=5.26 ± 3.81	.05	-.02	-.07	-.11**
Combat Experiences	15	#	0-60	P2=8.77 ± 6.85	-.00	-.05	-.00	-.09**
Unit Cohesion	3	P2=.91	3-15	P2=9.29 ± 3.04	.06	.01	.09	.19**
PTSD Checklist	17	P2=.93	17-85	P2=32.30 ± 11.50	-.07	-.17*	-.28*	-.53**
Physical Symptoms	15	P1=.76	0-30	P1=5.24 ± 4.02	-.33**	-.23**	-.48**	-.23**
		P2=.79		P2=8.69 ± 4.98	-.21**	-.34**	-.20**	-.40**
Injury in Theater	1	#	0-1	P2= 0.40± 0.49	--	--	--	--
SBP	--	--	--	P1=124.23 ±13.56	.04	.13**	.13**	.07
DBP	--	--	--	P1=72.86 ±11.20	-.02	.00	.17**	.11**
Δ SBP	--	--	--	P1=11.66 ±8.03	.07*	.17**	.07**	.07
Δ DBP	--	--	--	P1=7.20 ± 5.03	.07*	.14**	.04	.02

Correlations are between the measure (row) assessed at the phase listed in column 5 and the measure (column) assessed at the phase listed in the column title. ***Note. P1 = Phase 1, P2 = Phase 2, VR-36 = Veteran’s Rand-36, PCS = Physical Composite Score (Physical Health Function), MCS= Mental Composite Score (Mental Health Function), SBP=systolic blood pressure mean during three stressor tasks, DBP=diastolic blood pressure mean during three stressor tasks, Δ SBP=systolic blood pressure reactivity during three stressor tasks, Δ DBP=diastolic blood pressure reactivity during three stressor tasks, *= *P* <.05, **=P<.01 Numbers for P1 include the full sample (maximum N=790) and numbers for P2 are for the subsample with P2 data (maximum N=422).

# indicates a checklist measure, a single item or a variable for which no reliability coefficient can be calculated.

We used stepwise hierarchical linear regression models with variables added in three conceptually defined steps to arrive at two final multivariate models, one for physical function and one for mental function. In step one, we included the hypothesized demographic factors, and then used backward elimination of variables, one by one in subsequent steps, until only demographic variables that contributed significantly to the models were retained (i.e., those significant at p < 0.05 or better, after removing the variable with the highest p value one at a time). We next added the pre-deployment psychosocial and physiological factors, again with backward elimination of variables until only variables contributing significantly to the model were retained. Lastly, we added the deployment-related factors to the models, again with backward elimination until we arrived at final models of physical and mental function, respectively. BMI and smoking status were included as control variables for blood pressure and eliminated only when they and any blood pressure variable no longer contributed significantly to the overall model. Multicollinearity was assessed and variables were included only if the variance inflation factor was < 4 [30]. Missing data was handled using multiple imputation with imputed data generated using a sequential regression imputation method via the software package IVEware [31]. Multiple sets of imputed results were combined using Rubin’s rule which was implemented in SAS v9.2 MIANALYZE [31-33].

## Results

### Representativeness of the sample

The demographic characteristics of the sample, and a comparison with the demographics of the overall Army National Guard and Reserves, are shown in Table 2. Our sample was generally representative of the U.S. reservist component at the time of data collection, albeit with a somewhat larger proportion of males and Caucasian participants. More than half of the sample (56%) was deploying for the first time.

**Table 2:** **Comparison of this sample**, **and the overall Army National Guard and Reserves**

	**Our sample**	**Army National Guard (ARNG)/**
		**Army Reserve (AR)**
Age - Mean years (SD; range)	28.0 (8.3; 18–57 years)	Ages 18–60 years (ARNG overall Mean age approx. 33 years; Army Reserve enlisted average age is 31 years*)
Component		
Army NG	554 (72.2%)	Deployed reservist Army personnel are 71% ARNG & 29% Army Reserves +
Army Reserve	202 (26.3%)	
Active or Other	11 (1.4%)	
Males	688 (89.7%)	Army NG female = 13.4% Ŧ
Females	79 (10.3%)	Army Reserve female = 23.8% Ŧ
		Overall Army Res comp. = 17.6% Ŧ
Education – Mean years (SD)	97.4% were high school graduates or equivalent and 2.0% had bachelor’s degree or equivalent number of years.	In the Army NG and Army Reserve 99.8% and 99.3%, respectively were high school graduates or equivalent, and 15% had a bachelor’s degree*
Race		Army NG (%)/Army Reserves (%)
White	592 (77.2%)	
Black	69 (9.0%)	White 73.0 57.3
American Indian	21 (2.7%)	Black 13.9 23.2
Asian/Pac. Islander	21 (2.8%)	
Other	48 (6.3%)	Asian/Pac. Isl. 1.9 4.4
Declined/Missing	16 (2.1%)	Other 1.3 0.7
Ethnicity		Unknown 1.7 0.9
Hispanic	95 (12.4%)	Hispanic 8.2 13.5

*Average age and education for Reservists from Army Reserve Association, Inc. (2008) Specialized Workforce online http://www.armyreserve.org/SPECIALIZED_WORKFORCE.html.

+ Proportions based on numbers of Army NG and Army Reserve troops serving in Iraq and Afghanistan as of January 2, 2008. Waterhouse & O’Bryant (January 17, 2008) Congressional Research Service Report for Congress National Guard Personnel and Deployments: Fact Sheet (utilizing data from Department of Defense, Office of the Joint Chiefs of Staff, Legislative Affairs, January 2, 2008).

Ŧ Race/ethnicity data from September 2008 data; http://diversity.defense.gov/Resources/Commission/issue_papers.aspx; Issue Papers 54 and 55.

To assess non-participant bias, a subset of 243 individuals who declined to participate in the study were anonymously asked to report their gender and the initial item from the VR-36 [3] which asks respondents to rate their overall health. We dichotomized the general health question into excellent/very good vs. good/fair/poor. A Chi-square test showed no significant difference in the proportion of males and females in the participant and non-participant groups (*χ*^2^ =1.89, *P* = .17). However, participants were somewhat less likely than non-participants to report that they were in excellent/very good health (70% of participant sample vs. 76% of non-participant sample; *χ*^2^ = 4.98, *P* < .03).

### Outcome and psychosocial measures

In Table 1 we provide the Cronbach’s alpha reliability coefficients (where applicable), the means and standard deviations for the outcome and psychosocial measures used in our models, and correlations with physical and mental function at Phases 1 and 2.

Both physical and mental function showed a statistically significant decrease immediately following deployment relative to pre-deployment levels. On average, physical health function declined by 2.2 points from pre- to immediate post-deployment (t = 6.65, *P* <.0001), and mental health function declined by 4.0 points (t = 7.11, *P* <.0001). Considering only the subsample of individuals for whom we have data for both phases (i.e., unimputed data), we still see declines of 2.1 points (t = 6.14, *P* <.01) in physical function and 2.6 points (t = 4.93, *P* <.01) in mental function.

### Unadjusted relationship of predictors to physical and mental function

We examined the relationships between the continuous predictor variables and our two outcome variables at both Phases 1 and 2 (see Table 1) using Pearson’s correlation coefficients. Demographic variables that were related to poorer physical function included older age (r = −.19, *P* <.01), greater BMI (r = −.11, *P* <.05) and a larger number of previous deployments (r = −.13, *P* <.01). Psychosocial factors that were related bivariately to poorer physical function at Phase 2 included poorer physical function at Phase 1, more pre-deployment stressful life events, greater avoidance coping, less approach coping, more PTSD symptoms and greater physical symptom severity. Higher baseline Phase1 systolic blood pressure and blunted Phase 1 systolic and diastolic blood pressure reactivity to the stressor tasks also were related to poorer physical health function at Phase 2.

Demographic variables related to poorer mental health function included younger age (r = .20, *P* <.01) and lower BMI (r = .11, *P* <.01). Most of the hypothesized Phase 1 psychosocial and physiological variables were related to mental health function at Phase 2. The strongest bivariate predictors of poorer mental health function at Phase 2 were greater negative emotionality, greater avoidance coping, poorer mental health function at Phase 1, more PTSD symptoms, greater physical symptom severity, lower social support and lower social desirability.

Minority status was not related to either physical or mental function at Phase 1 or 2. Gender was not related mental health function at Phase 1 or physical health function at Phases 1 or 2, but females did have poorer mental health function than males at Phase 2 (diff = −3.73 (SE =1.68); p = .03). Having an injury was related to poorer physical health function at Phase 2 (diff = −3.78, SE =1.05); p = .00), but not related to physical function at Phase 1 or mental function at Phases 1 or 2.

### Adjusted predictors of physical and mental function

For the models predicting physical and mental function, all hypothesized variables with a significant bivariate relationship (at *P*<.10) with the outcome (physical or mental function) were entered into each of the two models as described above. Results after each step of each model are summarized in Tables 3 (physical function) and 4 (mental function).

**Table 3:** Models predicting phase 2 physical function

	**Model 1 R**^**2**^**=.05**				**Model 2 R**^**2**^**=.18**				**Model 3 R**^**2**^**=.26**			
	**b**	**SEMb**	**β**	**t**	**b**	**SEMb**	**β**	**t**	**b**	**SEMb**	**β**	**t**
Age	-.16	.04	-.17	-.3.63**	-.15	.04	-.16	-3.51**	-.15	.04	-.16	-3.81**
Body Mass Index	-.13	.09	-.09	1.52**	-.04	.08	-.03	-.50	-.03	.08	-.02	-.41
Gender	−2.43	1.19	-.10	-2.04*	--	--	--	--	--	--	--	--
Physical Function (Phase 1)					.37	.07	-.25	5.17**	.35	.07	.24	5.34**
Approach Coping					-.27	.13	-.10	-2.07*	--	--	--	--
Physical Symptoms (Phase 1)					-.28	.09	-.14	-3.13**	--	--	--	--
Smoking					.61	.78	.04	.79	.35	.66	.02	.52
Systolic Blood Pressure reactivity after a stressor					.14	.05	.15	3.13**	.11	.04	.12	2.58*
Physical Symptoms (Phase 2)									.38	.06	-.24	-5.79**
Injury									-2.46	.98	-.16	-2.50*

** *P* < .001, * *P* < .05. Each successive step accounted for a significant increase in variance over the prior model (all ps < .05 or better). Overall final model adjusted R^2^ = 0.26. For the model predicting physical health function at Phase 2 we included the following Phase 1 variables: gender, age, body mass index (BMI), number of previous deployments, physical health function at Phase 1, pre-deployment life events, approach coping, avoidance coping, physical symptoms, systolic blood pressure, systolic blood pressure reactivity to a stressor, and smoking. We also included the following Phase 2 variables: PTSD symptoms, physical symptoms and injury. Non-significant predictors were eliminated using a backward elimination method. Higher scores indicate better physical function.

The final model for physical function demonstrated that, controlling for the other predictors, poorer physical function at Phase 2 was associated with older age, poorer physical function at Phase 1, blunted systolic blood pressure reactivity to a lab stressor, greater physical symptom severity, and being injured.

The final model for mental function demonstrated that, controlling for the other predictors, poorer mental health function at Phase 2 was associated with younger age, lower social desirability, lower social support at Phase 1, poorer mental health function at Phase 1, more physical symptoms and more PTSD symptoms.

## Discussion

This study was designed to prospectively identify psychosocial and physiological factors associated with physical and mental function after deployment. On average, physical function in OEF/OIF soldiers declined by 2.2 points and mental function declined by 4.0 points from just before to just after a combat deployment. A two to three point difference on these measures is considered a minimal clinically important difference [16,34]. Thus, over the course of a year-long combat deployment, these individuals showed an average decline in physical and mental function that was meaningful, particularly in light of their young average age, and pre-deployment good health (compared to veteran norms). This is also notable in light of recent evidence showing that physical and mental function continue to decline over time since deployment [35-38]. This is the first prospective study to show the effects of combat deployment on health function. Moreover, it is important to identify, as we have done here, both pre- and early post-deployment factors that are associated with poorer physical and mental function to determine who is at greatest risk, and to determine which of these factors could be addressed by health care personnel or other caregivers.

We predicted that the effect of combat deployment on physical and mental function would be influenced by multiple demographic, psychosocial, physiological and deployment factors. After controlling for pre-deployment physical function, we found that poorer physical function after deployment was associated with older age, lower (i.e., blunted) systolic blood pressure reactivity to stressors, greater concurrent physical symptom severity, and being injured. In contrast, previous cross-sectional studies have suggested a variety of other factors to relate to physical function including PTSD diagnosis or symptoms [39,40], social support [41] and other psychosocial factors [42]. These previous studies often measured physical function many years after deployment raising the possibility that the effects of psychosocial factors occur over an extended time. Our findings do suggest that older Army enlisted personnel are at modestly greater risk for poor physical function after deployment. In our adjusted model (Model 3 in Table 3) soldiers showed a decrease in physical function of 0.15 units for every one year increase in of age leading to a clinically significant decline (2.5 point decrease) for every 17 years of age. Injured soldiers also showed a clinically significant decline in physical function (2.46 point difference) as compared to those with no injuries.

Blunted systolic blood pressure responses to laboratory stressors were associated with poorer physical health function after deployment. From one perspective, this may seem surprising given that greater cardiovascular responses to stressors have frequently been associated with poorer cardiovascular health [27]. However, recently several investigators have also demonstrated that blunted blood pressure responses to stressors can relate prospectively to poorer health outcomes, in particular depression, obesity, and overall poorer self-reported health [28].

Immediately after deployment these soldiers, on average, reported poorer mental function than before deployment. The final model, controlling for pre-deployment mental health function, showed that this lower mental health function was associated with younger age, lower social desirability, lower social support at pre-deployment, greater physical symptom severity, and having more PTSD symptoms. In our adjusted model (Model 4 in Table 4) we found a clinically significant difference in mental health function (i.e., 2.5 points) for every 15 years difference in age, but unlike for physical function, younger individuals were at greater risk for poor mental health function. Also unlike physical function, social support remained a significant predictor in the final model for mental health adding to a growing literature on the importance of social support both in- and out of the theater of war for OEF and OIF veterans [43,44]. We observed a 0.08 point change in mental function for every one point change in social support, or a clinically significant change in mental health function (2.5 point change) for about every 1.5 standard deviation (SD=18.9) difference in social support (Model 3 in Table 4). Thus, soldiers with poor social support may need additional resources prior to deployment or during deployment to buffer against declines in mental function. Moreover, soldiers reporting PTSD symptoms after deployment should also be assessed for declines in mental function. A clinically significant decrease in mental health function (2.5 point change) was associated with a 7.5 point increase in PTSD symptoms (Model 3 in Table 4). To provide context, the suggested cut off for a potential PTSD diagnosis on this measure is 33 points above the minimum score.

**Table 4:** Models predicting phase 2 mental function

	**Model 1 R**^**2**^**=.06**				**Model 2 R**^**2**^**=.23**				**Model 3 R**^**2**^**=.40**			
	**b**	**SEMb**	**β**	**t**	**b**	**SEMb**	**β**	**t**	**b**	**SEMb**	**β**	**t**
Age	.24	.06	.19	4.19**	.17	.05	.13	3.06*	.17	.05	.13	3.58**
Body Mass Index	15	.10	.07	1.40	--	--	--	--	--	--	--	--
Mental Health Function (Phase 1)					14.	.07	.12	2.12*	.14	.05	.12	3.02**
Social Desirability					.32	.14	.11	2.36*	.32	.12	.11	2.65**
Negative Emotionality					.08	.03	.11	2.97**	.08	.03	.13	2.76**
Social Support									-.38	.10	-.17	-63**
Physical Symptoms (Phase 2)									-.33	.04	-.35	-8.12**
Post-Traumatic Stress Disorder Symptoms (Phase 2)												

** p < .001, * p < .05. Each successive step accounted for a significant increase in variance over the prior model (all ps < .05 or better). Overall final model adjusted R^2^ = 0.40. For the model predicting mental health function at Phase 2 we included the following Phase 1 variables: gender, age, BMI, mental health function at Phase 1, social desirability, negative emotionality, absorption, social support, approach coping, avoidance coping, pre-deployment life events, alcohol misuse, physical symptoms, diastolic blood pressure at Phase 1 and smoking. We also included the following Phase 2 variables: deployment experiences, combat experiences, physical symptoms and PTSD symptoms. Non-significant predictors were eliminated using a backward elimination method. Higher scores indicate better mental function.

A strongest predictor of poorer physical and mental function was greater concurrent physical symptom severity. Physical symptoms are caused not only by the physical demands of combat, such as carrying heavy equipment, but also by psychosocial and environmental factors [45,46]. Scores on the physical symptom scale can be used to determine minimal (PHQ score = 0–4), low (PHQ score = 5–9), medium (PHQ score = 10–14) and high (PHQ score = 15–30) severity subgroups as suggested by Kroenke et al. [22]. Our adjusted data show a 0.38 point decline in mental and physical health function for every one point increase in physical symptoms, or alternatively a 5.7 point (i.e., double the minimal clinically significant change) difference between the lowest and highest symptom subgroups (Model 3 in Table 3). Clinicians should be aware that Veterans returning from a combat deployment with numerous or severe physical symptoms likely have significant impairments in mental and physical health function. Although physical symptoms were a primary focus of post-deployment health care for Persian Gulf War veterans (e.g., [47]) there has been much less emphasis on physical symptoms, broadly defined, and their impact on OEF/OIF veterans. In large part, this is because the literature on OEF/OIF Veterans has focused more on PTSD, mTBI (including associated physical symptoms often called post-concussive symptoms) and more recently on the co-morbidity of PTSD and/or mTBI with chronic pain (e.g., [48-50]). Our findings suggest that physical symptoms are an important contributor to both physical and mental function after war, over and above other factors, and thus may deserve additional focused attention.

This study was designed to address limitations of prior cross-sectional studies in combat veterans where many samples were treatment-seeking, assessments were obtained long after deployment, and/or there was a lack of pre-deployment data. This study has its own limitations, including the absence of a non-deployed comparison cohort, and loss-to-follow up due to a number of units and individuals who did not return to the stateside Army installations from which they deployed. To have a relatively healthy pre-deployment sample and to minimize confounds with our physiological variables, we also excluded from participation those participants with depression, schizophrenia, bipolar disorder or current hypertension which could have resulted in some range restriction on health function at baseline. However, respondents were somewhat more likely to report having poor general health than non-respondents, suggesting that any bias due to health exclusions may have been balanced by a modest tendency for those with poorer health to be more likely agree to participate. Although it does not currently include physiological measures such as those reported here, the large prospective epidemiological study, the Millennium Cohort Study [51] does not exclude potential participants for health conditions, and thereby will provide broadly generalizable results at least for self-report measures. In addition, collecting data at the mobilization site does not provide an optimal, non-stressful baseline. Social desirability, or the tendency to present oneself in an especially favorable way, was a significant predictor of mental function, as has been reported previously [52]. This suggests that like non-veterans, some military personnel present themselves as having better mental function than may in fact be true due to their biased reporting style. Another potential limitation is that for logistical reasons, we had to use different assessment methods (e.g., in-person computer surveys vs. in-person paper or phone surveys) at different time points which may have introduced additional measurement error.

In conclusion, this study sought to understand pre-deployment and early post-deployment predictors of physical and mental function immediately after deployment. We found that deployment was associated with a decline in both physical and mental function. The demographic, psychosocial, physiological and deployment experiences of OEF and OIF veterans are multifaceted and highly varied, and the effects of these factors on physical and mental function appear to be complex. Social support emerged as a potentially modifiable pre-deployment factor for mental function, and across both physical and mental function, increased physical symptom severity was an indicator of poorer post-deployment function even accounting for baseline function. These data suggest that health care providers should be aware that greater physical symptom severity, lower social support, more PTSD symptoms and having had a deployment injury all are important clinical features that may suggest the need for further functional evaluation for a veteran returning from a recent combat deployment to Iraq or Afghanistan.

## Competing interests

The authors declare that they do not have any conflicts of interest.

## Authors’ contributions

LMM collected data, designed the analysis plan, wrote the initial draft and contributed extensively to the writing; ED collected data and contributed to the writing; SL conducted the data analysis and contributed to the writing, BA contributed to the writing, GY contributed to the writing, CE contributed to the study design and the writing, KQ designed the study, collected the data, designed the analysis plan, and wrote parts of the initial draft and contributed extensively to the writing. All authors read and approved the final manuscript.
